# Supplementary material for: KOMPEITO, an Atypical Arabidopsis Rhomboid-Related Gene, Is Required for Callose Accumulation and Pollen Wall Development
Source: Int J Mol Sci. 2022 May 25;23(11):5959. doi: 10.3390/ijms23115959 (PMC9180352; doi:10.3390/ijms23115959)
Supplement: Supplementary file 1 [file ijms-23-05959-s001.zip › ijms-1684704-supplementary.pdf]

International Journal of
Molecular Sciences

Supplementary Methods

Microscopy

Samples for ovule observations were prepared in the same manner as for pollen observations (see Materials and Methods). For observations of pollen tubes and megaspore mother cells, pistils were first fixed, treated with 90% and 70% ethanol for 20 minutes each, and then treated with 1 N NaOH overnight. The pistils were stained in 0.1% aniline blue in 0.1% K_3PO_4 buffer (pH approximately 12.4) for 1 hour, mounted in glycerol and observed under UV illumination. For DAPI staining, pollen grains were dipped into DAPI staining solution (2 μg/ml DAPI in 7% sucrose), stained for 15 minutes and observed under UV illumination.

Ligand cleavage assay for Rhomboid proteases

A PCR product of *KOM* cDNA was cloned into pcDNA 3.1(-) (Invitrogen, Carlsbad, CA). A triple HA tag was inserted after the initiator methionine of KOM. HA-tagged Rhomboid-1, and GFP-tagged Spitz, Keren, and TGFα constructs, and ligand cleavage assay conditions for Rhomboid proteases was performed as described elsewhere [1-3].

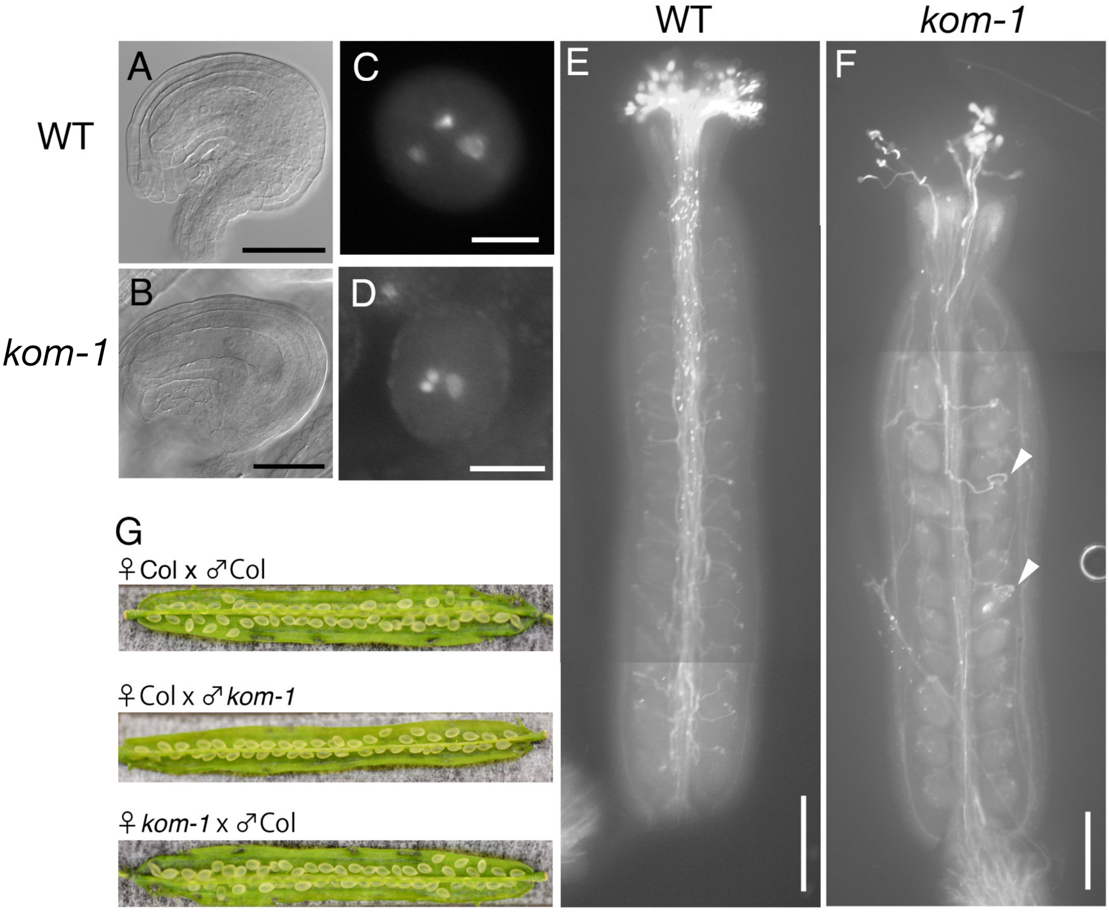

Figure S1. Development of male and female gametophytes in *kom-1*. *(A, B)* Ovule development in *kom-1 (B)* proceeded as normally as in WT *(A)*. *(C, D)* DAPI staining of mature pollen grains. Three nuclei, including two generative and one vegetative, are visible in WT *(C)* as well as in *kom-1 (D)*, indicating that the *kom-1* mutation does not affect male gamete development. *(E, F)* Pollen tube growth inside pistils. Less pollen grains of *kom-1* could stay adhered to the stigma. However, once germinating, they developed normal pollen tubes inside pistils (arrowheads) and could fertilize female gametes *(F)* as compared to WT *(E)*. *(G)* Cross-pollination experiment. Col pistil pollinated with *kom-1* pollen (middle) and *kom-1* pistil pollinated with Col pollen (bottom) resulted full seed production, which is similar to Col self-pollination (top). Bars: *A, B*, 50 µm; *C, D*, 10 µm; *E, F*, 500 µm.

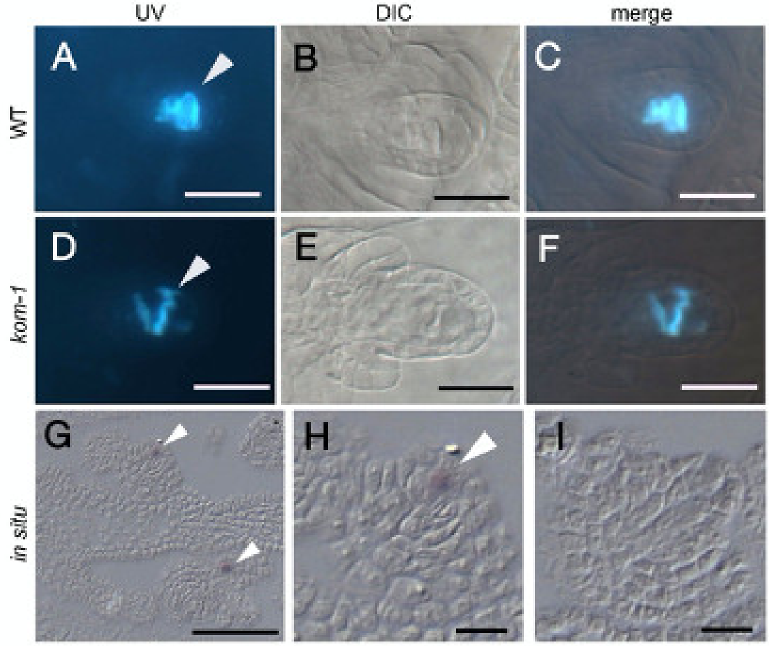

Figure S2. Effect of the *kom-1* mutation on callose accumulation during megasporogenesis, and analysis of *KOM* expression in pistils. *(A-F)* Aniline blue staining of developing megaspores. Callose was present in callose walls (arrowheads) surrounding megaspore mother cells in both WT *(A)* and *kom-1 (D)*, but the amount of callose was reduced in *kom-1*. No apparent defect in the development of megaspore mother cells could be observed using light microscopy in *kom-1 (E)* as compared to that in WT *(B)*. Images of *(A)* and *(B)*, and *(D)* and *(E)* were superimposed to generate *(C)* and *(F)*, respectively. *(G-I)* Analysis of *KOM* expression in pistils by *in situ* mRNA hybridization. *KOM* expression was detected in megaspore mother cells undergoing meiosis (arrowheads) *(G)*. Higher magnification of *(G)* is shown in *(H)*. Sense mRNA of *KOM* was used as a negative control *(I)*. Bars: *A-F*, 50 μm; *G*, 100 μm; *H, I*, 20 μm.

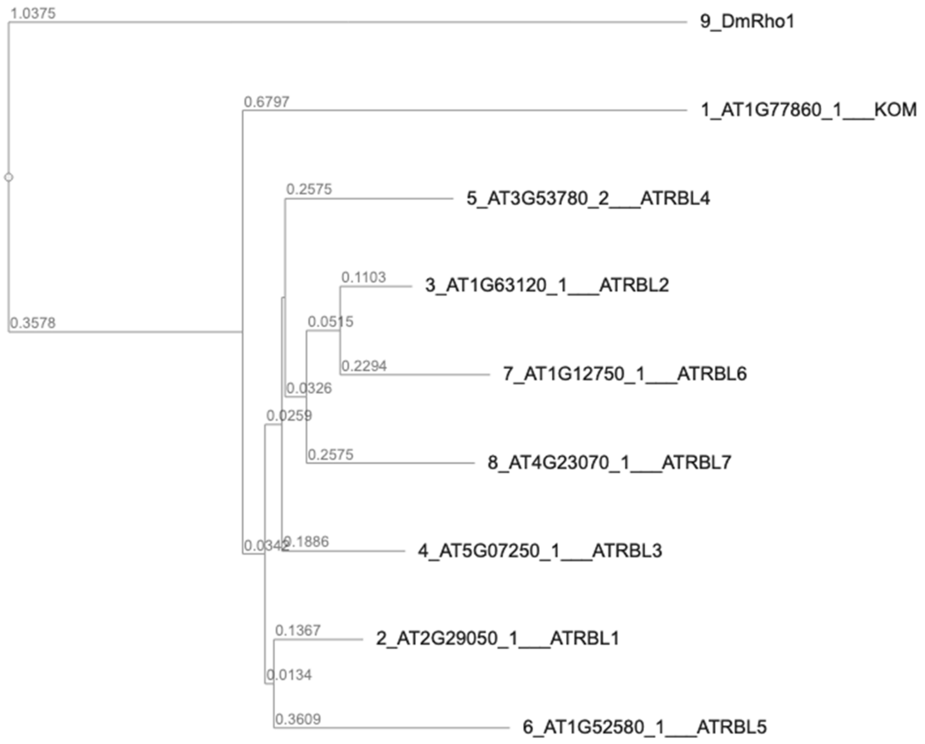

Figure S3. Phylogenetic analysis of Arabidopsis Rhomboid proteins. Drosophila Rhomboid-1 was used as an outgroup. Neighbor-Joining method was performed to show KOM is a distantly-related member of Arabidopsis Rhomboid proteins.

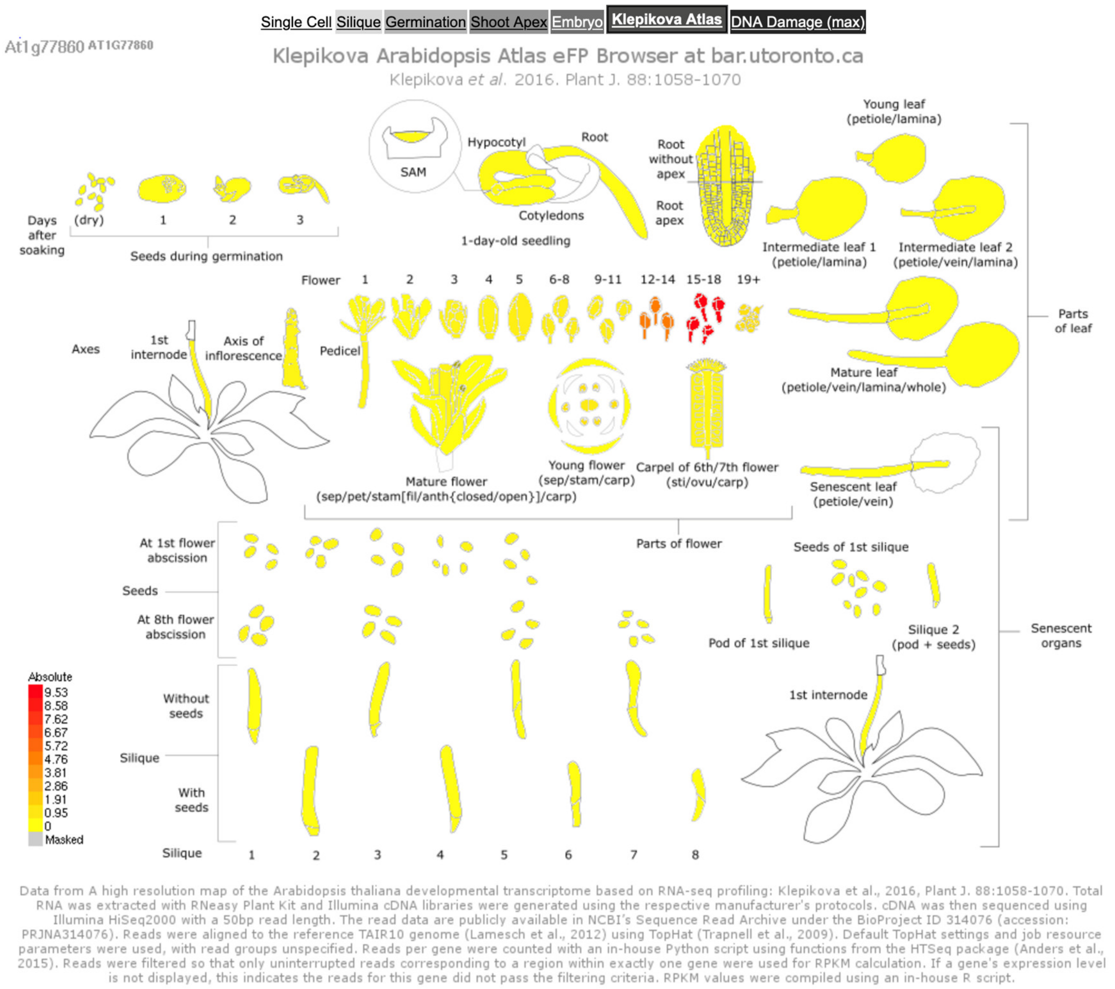

Figure S4. Expression of *KOM* gene. RNAseq data for *At1g77860* (*KOM*) gene were obtained from (http://bar.utoronto.ca/efp/cgi-bin/efpWeb.cgi).

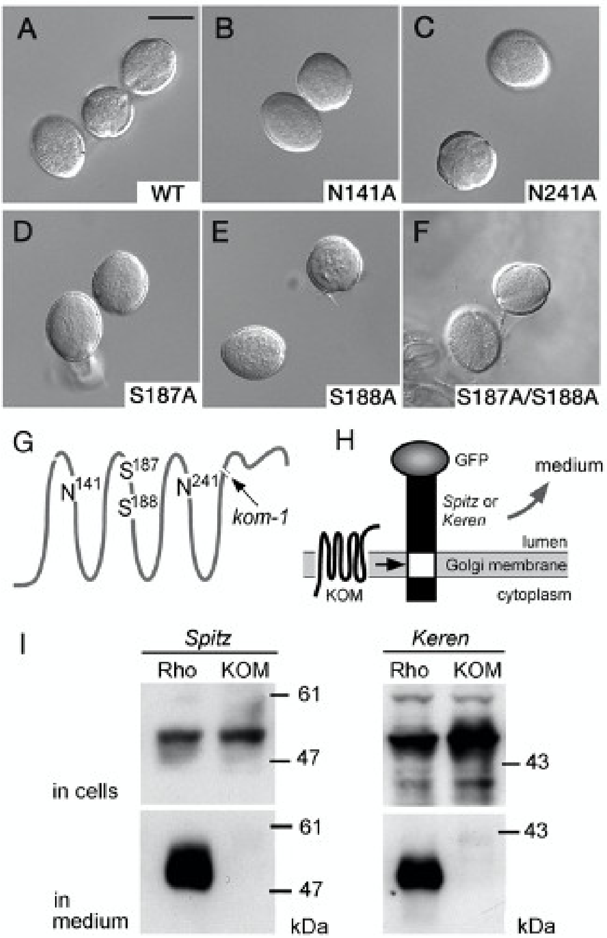

Figure S5. KOM is not a typical Rhomboid. (A-G) Complementation of *kom-1* with point-mutated *KOM* constructs. Residues N^{141} in TMD2, S^{187} and S^{188} in TMD4, and N^{241} in TMD6 are highly conserved during evolution and are required for the protease activity of Drosophila Rhomboid-1. The *kom-1* mutation results in the synthesis of a defective truncated protein lacking the C-terminal 70 amino acids (G). Transgenic *kom-1* plants expressing point-mutated *KOM* genes, N141A (B), N241A (C), S187A (D), S188A (E) and S187A/S188A (F), produced normal pollen grains which were morphologically indistinguishable from that in WT (A). (H-I) Ligand cleavage assay for KOM. KOM was co-expressed with GFP-tagged Spitz and Keren (as a substrate) and Star in mammalian COS cells. Drosophila Rhomboid-1 (Rho) served as a positive protease control and was able to cleave the substrate in the transmembrane domain, releasing the N-terminal portion of GFP-tagged Spitz and Keren from the cells into the medium. (H). The substrates inside COS cells was detected on Western blots using anti-GFP polyclonal antibodies. Processed products of Spitz and Keren were not detected in the medium when KOM was expressed (I). Bar in A: 20 μm for A-F.

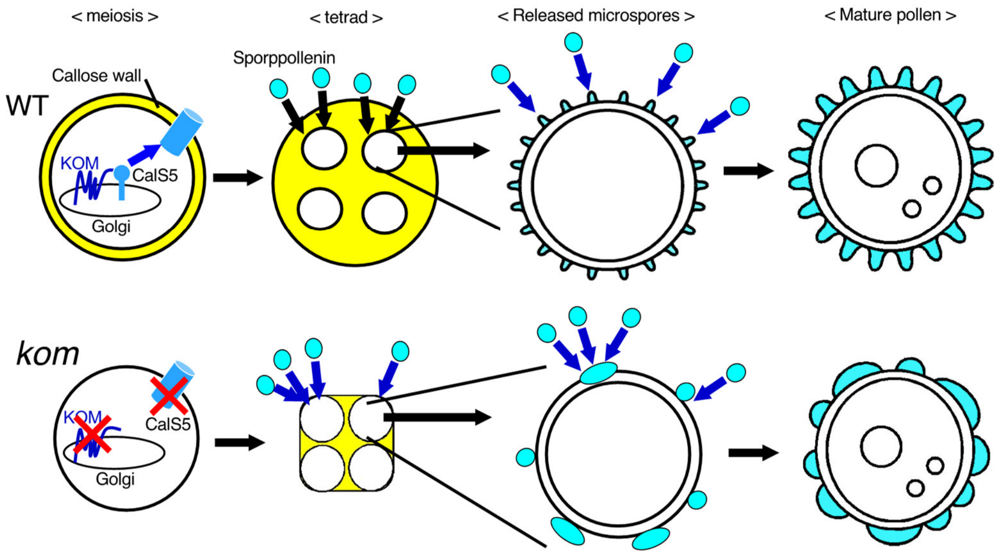

Figure S6. Schematic motel of KOM for callose and pollen wall formation. In WT, KOM localizes Golgi apparatus. CalS5 callose synthase protein is transported to plasma membrane through secretory pathway to produce callose wall. Sporopollenin is deposited onto the surface of microspores and makes typical pattern. In *kom* mutant, the amount of CalS5 protein is reduced so callose wall is not properly formed. Sporopollenin deposition is also perturbed, leading to form irregular pollen wall pattern.

References

1. Lee, J.R.; Urban, S.; Garvey, C.F.; Freeman, M. Regulated Intracellular Ligand Transport and Proteolysis Control EGF Signal Activation in Drosophila. *Cell* **2001**, *107*, 161–171, https://doi.org/10.1016/s0092-867400526-8.
2. Urban, S.; Schlieper, D.; Freeman, M. Conservation of Intramembrane Proteolytic Activity and Substrate Specificity in Prokaryotic and Eukaryotic Rhomboids. *Curr. Biol.* **2002**, *12*, 1507–1512, https://doi.org/10.1016/s0960-982201092-8.
3. Urban, S.; Lee, J.R.; Freeman, M. Drosophila Rhomboid-1 Defines a Family of Putative Intramembrane Serine Proteases. *Cell* **2001**, *107*, 173–182, https://doi.org/10.1016/s0092-867400525-6.
